# Barriers and Solutions to Implement HIV Pre‐Exposure Prophylaxis Program Among Key Populations in Iran: A Qualitative Study Using Causal Layered Analysis

**DOI:** 10.1002/hsr2.70968

**Published:** 2025-07-07

**Authors:** Hossein Moameri, Parya Saberi, Najaf Ahmadi Aghziarat, Parvin Mangolian Shahrbabaki, Reza Goudarzi, Mohammad Mehdi Gouya, Ali Mirzazadeh, Hamid Sharifi

**Affiliations:** ^1^ Student Research Committee, School of Public Health Kerman University of Medical Sciences Kerman Iran; ^2^ HIV/STI Surveillance Research Center, and WHO Collaborating Center for HIV Surveillance, Institute for Futures Studies in Health Kerman University of Medical Sciences Kerman Iran; ^3^ Division of Prevention Science University of California San Francisco San Francisco California USA; ^4^ Department of Biostatistics and Epidemiology, Faculty of Public Health Kerman University of Medical Science Kerman Iran; ^5^ Department of Medical‐Surgical Nursing, Physiology Research Center, Razi School of Nursing and Midwifery Kerman University of Medical Sciences Kerman Iran; ^6^ Health Services Management Research Center, Institute for Futures Studies in Health Kerman University of Medical Sciences Kerman Iran; ^7^ Iran University of Medical Sciences Tehran Iran; ^8^ Department of Epidemiology and Biostatistics University of California San Francisco California USA; ^9^ Institute for Global Health Sciences University of California, San Francisco San Francisco California USA

**Keywords:** causal layered analysis, Iran, key populations, pre‐exposure prophylaxis

## Abstract

**Background and Aims:**

HIV pre‐exposure prophylaxis (PrEP) has a crucial role in HIV prevention globally. Our study aimed to explore the approaches to address and overcome barriers to PrEP implementation among key populations in Iran.

**Methods:**

In this multi‐stage qualitative study, first, we employed in‐depth interviews to identify possible barriers to PrEP implementation in Iran. Second, we conducted three focus group discussions with HIV experts to categorize barriers to PrEP implementation among key populations using the Causal layered analysis. Last, we collected HIV expert's opinions on possible solutions to overcome the barriers.

**Results:**

We found four layers of barriers (including potential solutions) in PrEP implementation: litany, systemic causes, and discourse/myth. In the litany layer, increasing HIV knowledge among key populations and healthcare providers was identified as a solution. In the systemic layer, cooperation and coordination among all societal organizations and providers were reported as a solution. In the discourse/myth layer, gaining the trust of the key populations, providing PrEP in private sectors, using telemedicine, and religious beliefs were identified as solutions.

**Conclusion:**

Our deep multi‐step qualitative analysis showed that barriers to implementing PrEP in Iran are several, complex, and cross‐cutting. Addressing them requires interventions among key populations at risk for HIV, such as PrEP users, health providers, and the community in general, to build a supportive environment for PrEP as one of the HIV prevention strategies in Iran.

## 1 Introduction

While behavioral strategies have had an important effect on reducing new HIV infections [1, 2], the latest statistics show that over 39 million people were living with HIV in 2022 worldwide [3]. In Iran, nearly 2900 new HIV cases were reported in 2022 [4]. These numbers show that current HIV prevention measures, including traditional behavioral strategies, are insufficient for addressing the epidemic [5]. Effective interventions must be considered to reduce the number of infections, such as adding biomedical interventions [6].

HIV pre‐exposure prophylaxis (PrEP) has the potential to alter the course of the HIV epidemic significantly [7]. In 2012, the World Health Organization (WHO) highlighted the daily use of antiretrovirals to prevent HIV in HIV‐uninfected people [6]. Over the last decade, much progress has been achieved in implementing PrEP, and it is now used in many countries [8].

Most countries have encountered numerous barriers and challenges to implementing PrEP programs [9, 10]. Identifying, understanding, and finding solutions to reduce or eliminate these barriers is vital for successfully implementing PrEP [8]. However, PrEP is not implemented as part of Iran's HIV national prevention strategy, and no information about this intervention is available. As a result, before implementing this program in Iran, it is necessary to identify its challenges and barriers and present appropriate solutions. Understanding and addressing the complex barriers of PrEP implementation requires multiple approaches [11].

Causal layered analysis (CLA) has been proposed to investigate the layers producing problems and their solutions [12]. In this approach, the cause of an event is investigated in four layers [13]. Unlike traditional analytical methods that often focus on surface‐level trends or readily quantifiable data, CLA explores the deeper, usually implicit, layers of reality shaping our perceptions and actions. This method facilitates a more nuanced and comprehensive understanding, uncovering the interconnectedness of various factors and encouraging the development of more effective and sustainable solutions. Ultimately, utilizing CLA strengthens the rigor and depth of scholarly inquiry, enabling researchers to move beyond descriptive analysis toward a more critical and future‐oriented examination of complex challenges. The current study used the CLA approach to categorize barriers and find solutions for PrEP implementation among key populations in Iran.

## 2 Methods and Materials

This was a multi‐stage study to identify solutions for successful PrEP implementation among key populations in Iran. Addressing challenges in PrEP implementation among target populations necessitates a multifaceted approach to identify and analyze the underlying issues. Initially, we employed in‐depth qualitative interviews (key populations and HIV experts) to identify possible barriers to PrEP implementation in Iran. In the second stage, two focus group discussions (FGD) with HIV experts were conducted to categorize these barriers using the CLA framework. In the last step, HIV experts suggested potential solutions to address the barriers. In the last step, HIV experts discussed possible strategies and interventions to address the identified barriers in the third FGD. We also used the Consolidated Criteria for Reporting Qualitative Research (COREQ) in this study [14]. The COREQ improved the study's clarity and reproducibility, facilitating readers' better understanding of the research context, methodologies, and concepts.

### 2.1 Participants

Participants were selected through a purposeful sampling approach based on predetermined criteria. Eligibility criteria include a desire to participate in the study, ≥ 18 years old, HIV‐negative by self‐report, having Iranian citizenship, and at least one of the following criteria: (1) having had a sexual relationship to receive money or other service in the last 12 months for female sex workers (FSW), (2) having at least one sexual intercourse with other men for men who have sex with men (MSM), (3) injected any illicit substances for people who inject drugs (PWID), or (4) sexual partners of people living with HIV (PLHIV). We also included infectious disease specialists, university professors with experience working in the field of HIV, staff from the HIV control department, and healthcare workers.

### 2.2 Data Collection

A semi‐structured interview guide was used to identify the barriers to implementing PrEP. In‐depth face‐to‐face interviews were conducted for data collection by a trained gender‐matched interviewer in a private room. For interviews with HIV experts when they were not available for in‐person interviews, we conducted interviews virtually. The interviews continued until no new codes were added to our findings.

### 2.3 Data Analysis

We performed content analysis to identify possible barriers to PrEP implementation in Iran [15]. Moreover, the CLA framework was used to identify solutions for successful PrEP implementation [12]. The initial step involved the transcription of interviews in a word‐for‐word manner. Subsequently, the interview text was segmented into compact semantic units, condensing related words and phrases into each unit of meaning. These codes were then grouped into categories according to their similarities and distinctions. A category class at the manifest level consists of related code. After finding the meaning and content of the data, themes were created to express the underlying meaning of the text [15]. Categorizing barriers to successful PrEP implementation among key populations in Iran was done using the CLA framework. The last step discussed possible strategies and interventions to address the identified barriers. Based on the CLA strategy, the first layer, called the litany, analyzes the most superficial data, representing the official and accepted view of reality. The second layer, structural causes, represents the organized points of view. The third layer focuses on the analysis of worldview and discourse. This layer analyzes unconscious argumentation assumptions based on ideologies and worldviews. Finally, the fourth layer indicates the subject's unconscious motivating qualities as embodied in the form of myth [13]. Because of the subject being studied, we considered the third and fourth layers as one layer. Also, data were analyzed manually using Microsoft Word.

### 2.4 Ethical Considerations

The Ethics and Research Committee of Kerman University of Medical Sciences reviewed and approved the study protocol (Code: IR.KMU.REC.1401.443). Informed consent was obtained from participants before initiating and recording the interviews. Moreover, the unwillingness of participants to participate in the study did not affect their access to healthcare services.

## 3 Results

The analysis included 39 participants who were members of key populations or HIV experts. Of the 21 people in key populations, 7 were FSWs, 6 were MSM, 4 were PWID, and 4 were sexual partners of PLHIV. In addition, of the 18 people in the HIV expert group, 3 were infectious disease specialists, 4 were university professors with experience working in the field of HIV, 4 were staff from the HIV control department, and 7 were healthcare workers (Table 1). The identified solutions were categorized into three layers: litany (7 solutions), systemic causes (7 solutions), and discourse/myth (8 solutions) (Table 2).

**Table 1:** Demographic characteristics of participants.

Variable	Age (year), mean ± SD; (Minimum ‐Maximum)	Sex, *N* (%)
HIV experts^a^	51.4 ± 8; (37–68)	Female: 10 (55.5) Male: 8 (44.5)
Key population^b^	38.4 ± 8; (25–55)	Female: 9 (42.9) Male: 12 (57.1)

a Infectious disease specialists, university professors with experience working in the field of HIV, staff from the HIV control department, and healthcare workers.

b MSM, FSW, PWID, and sexual partners of PLHIV.

**Table 2:** Layer analysis of solutions to remove the barriers of PrEP among key populations in Iran.

	Barriers	Solutions
Litany	Lack of HIV knowledge among key populations	Effective training for key populations before starting PrEP^a^
	Lack of HIV knowledge among healthcare workers	Effective training for healthcare workers
	Inappropriate behavior of people in society with key populations	Increasing awareness of HIV and prevention knowledge in society HIV and anti‐stigma education in professional schools
	Economic challenges among key populations	Insurance coverage for key populations providing free PrEP
	Incapability to identify the key populations	Involvement of mobile outreach teams The existence of communication networks (such as WhatsApp, Instagram, etc.) to identify key populations
Systemic causes	Inadequate health services and incorrect service distribution management	Creating hotspot maps and giving PrEP in these locations Using HIV experts' experience to identify and implement key activities to address field problems Providing PrEP in accessible places for key populations (home and drugstore)
	Challenges in decision‐making	Providing a comprehensive report of HIV condition in the country to increase insight and help policymakers make more accurate decisions
	Internal and external inconsistencies in the organization	Creating guidelines for prescribing and monitoring PrEP by health workers Establishing a committee to collaborate with other organizations for improved implementation of PrEP
	Fear of legal treatment of key populations by law enforcement agencies	Justification of other organizations for security support of key populations
Discourse/Myth	Fear of stigma	The peer group collaboration Providing PrEP in the private sector due to less stigma for key populations Implementation of telemedicine regarding the provision of PrEP to reduce stigma
	Lack of trust in PrEP drugs	Using people living with HIV experiences to encourage PrEP use Changing misconceptions among key populations by expert counselors
	Insufficient trust in the health system Belief in the unchangeability of fate	Providing PrEP in private sectors due to the trust of these sectors by key populations Using the experiences of individuals living with HIV to promote trust in the healthcare system Using religious beliefs and knowledge of the changeability of fate

a Pre‐exposure prophylaxis.

Increasing HIV awareness and knowledge among the key population was identified as an important solution in the litany layer. Enhancing knowledge, especially using social media, was introduced as an important component of overcoming barriers. A 36‐year‐old man who has sex with men said, “*I think it would be great if information regarding this method (PrEP) were provided. The healthcare staff should provide this information. Because of their experience, they can provide us with reliable and precise information regarding this method. With this approach, more people would come to take the drug.”* In addition, the president of the VCT Center, with 20 years of experience, said, *“These groups are frequently well‐connected on social media. Previously, we employed this strategy, for example, in a location where we would conduct a test (HIV test). They would notify others rapidly through these networks. They would notify each other swiftly if anything happened. Therefore, these media could be used to inform and raise people's awareness.”*

In the systemic causes layer, maximal collaboration among organizations was identified as crucial to ensure that all stages, from needs assessment to the procurement and equitable distribution of PrEP, were implemented in coordination with all relevant public health institutions. For example, using peer support groups was one of the potentially effective solutions that was suggested for increasing people's desire to receive HIV prevention services. A technical officer from the national HIV program, with 15 years of experience, said, *“Look, the health system is capable of far more than only internal and external coordination. For example, peer groups can be used to share messages and information. In some groups, these people are very influential. If they are justified, they have a strong influence on others. They are more trusted in these communities.”* Additionally, a university faculty member with 12 years of experience in the HIV Department said: *“I believe that knowledge should be transferred from peer groups, particularly those who are well‐known and influential. Narcotics Anonymous is currently the world's best and most successful group for reducing drug abuse and quitting. I believe this is a beneficial experience that may be applied to other things, such as PrEP.”*

In the discourse/myth layer, establishing trust among key populations through varied approaches was identified as one of the most important solutions. Changing the misconceptions of people about HIV and PrEP was noted as necessary. Additionally, providing PrEP in the private sector due to higher trust among key populations and implementing telemedicine for PrEP provision to mitigate stigma were noted as potentially effective solutions. Furthermore, religious beliefs and a deep belief in the ability to change fate may be potential solutions for incorrect beliefs.

A policy‐maker with 7 years of experience working in the HIV department said, “*Look, these individuals think the system is inefficient or that they don't want to come here to be recognized. Because they might be familiar to locals. On the other hand, they go to the private sector and claim they obtain more and better‐quality services, but no one recognizes them because private sector statistics are not available anywhere.”* Additionally, a university faculty member with 15 years of experience working in the HIV field said, *“We do not have the essential infrastructure for these groups; our centers are limited, and they have a stigma. Generation Z, who are currently entering the high‐risk group, is unfamiliar with these centers and is accustomed to online and remote services. They buy their sandwiches online. In this situation, adopting remote services is beneficial.”*

## 4 Discussion

In this study, we explored several approaches to potentially address the barriers and challenges with PrEP implementation programs in Iran. Increasing awareness and knowledge and the optimal use of social media were identified as an important solution in the litany layer. In the systemic layer, the coordination of internal and external organizations and providing convenient access to PrEP services were noted as important factors. Providing PrEP in the private sector and by peer groups was suggested in the discourse/myth layer. Also, telemedicine implementation for the provision of PrEP to reduce stigma was noted to be a possible effective solution. The use of religious beliefs may be a potential solution for incorrect beliefs.

The need for increasing knowledge, which was identified in the litany layer, is not only needed among key populations but also in healthcare workers and the general population given their lack of knowledge about HIV and prevention strategies. Increasing HIV knowledge, prevention strategies, and awareness of existing interventions have the potential to significantly affect PrEP uptake among key populations [16]. A study revealed that willingness to initiate PrEP among MSM was associated with prior PrEP awareness [17]. Also, previous studies have shown that service providers did not have the necessary knowledge of PrEP [18]. As in our study, in prior studies, women have stressed the necessity of social media strategies to increase PrEP awareness [19]. Also, previous studies have shown that peer support groups and social media are effective methods that can increase HIV‐related knowledge [20, 21, 22]. As a result, by increasing the knowledge of key populations through community‐accepted methods, it is possible to reduce the barriers to initiating PrEP.

Intra‐departmental and interdepartmental coordination and appropriate planning and organizational solutions, such as the existence of comprehensive guidelines and providing PrEP in places of access to key populations, were identified as important solutions in the systemic layer. Previous studies have also identified the need for detailed guidelines including implementation steps [18] and free and easy‐to‐access PrEP [23]. Additionally, a systematic review showed that increased human resources are needed for the implementation of PrEP because, starting a PrEP service requires additional staff and testing equipment, thereby adding strain to the current healthcare system [18]. Key populations are frequently denied their basic right to healthcare [24]. Therefore, interorganizational coordination is critical not only for adequate PrEP access and HIV prevention but also for general healthcare and social needs.

The need to change the misconceptions of key populations and the general public about HIV, providing PrEP in private sectors and through peer groups, and implementing telemedicine were important strategies identified at the worldview level. Published studies have shown that false beliefs about PrEP by patients should be taken seriously and that the change of these views can strongly be influenced by the knowledge and beliefs of their service providers [25]. Moreover, in line with our study, studies in other countries have shown that innovative models to access PrEP through the private sector are beginning to emerge [26]. In addition to the provision of peer groups has been identified as an important solution due to their trust in the key populations [27, 28]. In addition, telemedicine can have an important impact on PrEP by reducing transit time and cost, reducing potential stigma in seeking care, and reducing in‐person medical visits [29, 30]. Educating individuals on the uncertainty of fate and its potential for change through timely and effective interventions is the solution to incorrect beliefs. As a result, due to the easy access and potentially lower stigma of the mentioned methods, it is possible to gain people's trust, which may provide access to communities experiencing discrimination, stigma, and transphobia. In addition, In addition, it may be important to change the beliefs of people using stories or methods that they are familiar with.

Given the very slow roll‐out of PrEP in Iran, it is unknown what other barriers and challenges may exist that are not discussed in this paper. Therefore, more advocacy for increased PrEP and further longitudinal research are needed to explore barriers.

## 5 Conclusion

Prioritizing and addressing the identified barriers is important to enable the future accessibility, acceptability, and feasibility of PrEP programs in Iran. However, different communities may require different solutions, and even within a society, varied solutions must be identified based on the root causes and degrees of the problems. Some solutions may need to be addressed using deeply held beliefs of individuals and groups.

## Author Contributions

**Hossein Moameri:** investigation, writing – original draft. **Parya Saberi:** conceptualization, writing – review and editing, validation. **Najaf Ahmadi Aghziarat:** investigation, methodology. **Parvin Mangolian Shahrbabaki:** methodology, conceptualization, visualization, project administration. **Reza Goudarzi:** validation, methodology, writing – review and editing. **Mohammad Mehdi Gouya:** validation, methodology, conceptualization. **Ali Mirzazadeh:** formal analysis, methodology, conceptualization, writing – review and editing. **Hamid Sharifi:** methodology, conceptualization, project administration, supervision, writing – review and editing, funding acquisition, validation.

## Conflicts of Interest

The authors declare no conflicts of interest.

## 1 Transparency Statement

The lead author Hamid Sharifi affirms that this manuscript is an honest, accurate, and transparent account of the study being reported; that no important aspects of the study have been omitted; and that any discrepancies from the study as planned (and, if relevant, registered) have been explained. All authors have read and approved the final version of the manuscript. The senior and corresponding author, Hamid Sharifi, had full access to all of the data in this study and takes complete responsibility for the integrity of the data and the accuracy of the data analysis.

## Data Availability

All data generated or analyzed during this study are included in this published article.
